# Histopathological Diagnosis of Pulmonary Paragonimiasis: A Case Report from Ecuador

**DOI:** 10.3390/tropicalmed11100264

**Published:** 2026-09-24

**Authors:** Katherine Fuentes-Ortiz, Ligia Gabriela Velalcázar-Oñate, Bruna Espín-Nuñez, Manuel Calvopiña

**Affiliations:** 1 Department of Pulmonology, Hospital de Especialidades Eugenio Espejo, Quito 170136, Ecuador; katherine.fuentes@hee.gob.ec; 2 Escuela de Postgrado, Doctorado en Ciencias de la Salud, Instituto Universitario Italiano de Rosario (IUNIR), Rosario S2000CTT, Argentina; gabrielavelalcazar@yahoo.es; 3 Department of Radiology and Diagnostic Imaging, Hospital de Especialidades Eugenio Espejo, Quito 170136, Ecuador; 4 Department of Pathology, Hospital de Especialidades Eugenio Espejo, Quito 170136, Ecuador; mdbrunaespin@gmail.com; 5 One Health Research Group, Universidad de las Américas (UDLA), Quito 170124, Ecuador

**Keywords:** pulmonary paragonimiasis, foodborne, diagnosis, lung biopsy, histopathology, Ecuador

## Abstract

Paragonimiasis is a foodborne trematode infection caused by *Paragonimus* spp. that primarily affects the lungs and may mimic tuberculosis, pulmonary mycoses, abscess, or malignancy because of its nonspecific clinical and radiological manifestations. Definitive diagnosis relies on the identification of *Paragonimus* eggs in respiratory specimens or tissue. We report a 17-year-old female from the Ecuadorian Amazon with a 5-year history of chronic cough and recurrent hemoptysis. Repeated examinations of sputum and stool were negative for *Paragonimus* eggs, and GeneXpert MTB/RIF assays and mycobacterial cultures were negative. Chest computed tomography performed approximately 1 year apart demonstrated a persistent 12 × 13-mm mixed solid-cystic lesion with central necrosis in the left upper lobe. Marked peripheral eosinophilia and elevated serum IgE were present. Bronchoalveolar lavage showed no parasitic structures, and a CT-guided percutaneous lung biopsy was nondiagnostic. Video-assisted thoracoscopic resection was subsequently performed. Histopathological examination revealed eosinophil-rich granulomatous inflammation and multiple thick-shelled, operculated eggs that were morphologically consistent with *Paragonimus* spp. Species-level identification was not performed. A detailed epidemiological history disclosed frequent consumption of raw freshwater crabs and shrimp. Following surgical resection and praziquantel treatment, respiratory symptoms resolved and eosinophilia markedly decreased; the patient remained asymptomatic during 1 year of follow-up. This case emphasizes that pulmonary paragonimiasis should be considered in patients from endemic areas with chronic hemoptysis, eosinophilia, compatible pulmonary lesions, and relevant dietary exposure, even when conventional parasitological examinations are negative. We highlight the value of histopathological examination of adequately sampled lung tissue when conventional parasitological investigations are repeatedly negative.

## 1. Introduction

Paragonimiasis is a foodborne zoonotic trematode infection caused by species of the genus *Paragonimus* and is recognized by the World Health Organization as a neglected tropical disease [1]. Infection is acquired through the consumption of raw or undercooked freshwater crabs or crayfish harboring infective metacercariae. The metacercariae excyst in the intestinal tract, penetrate the intestinal wall, and migrate through the peritoneal cavity, diaphragm, and pleural cavity before reaching the lung. Adult flukes primarily mature in the lung parenchyma, where they induce chronic inflammatory nodules and cystic or cavitary lesions and may survive for many years if left untreated [2]. Ecuador is endemic for *P. mexicanus* in both the Amazon and Coastal tropical ecoregions [3,4].

Pulmonary paragonimiasis is the most common clinical manifestation. However, overlapping clinical and imaging features with other pulmonary diseases make differential diagnosis challenging. Patients may present with chronic cough, rusty or blood-tinged sputum, recurrent hemoptysis, pleuritic chest pain, fever, and/or manifestations related to pleural effusion as pleuritic chest pain and dyspnea. Because of its insidious clinical course and nonspecific radiological findings, pulmonary paragonimiasis is frequently misdiagnosed as pulmonary tuberculosis, bacterial pneumonia, lung abscess, chronic pulmonary mycoses, or primary lung malignancy [5,6]. Hence, establishing the correct diagnosis is particularly challenging in regions where paragonimiasis coexists with other endemic pulmonary diseases. Extrapulmonary paragonimiasis occurs when *Paragonimus* parasite larvae migrate to organs outside the lungs, such as the brain, abdomen, or skin.

Chest radiography may show nodules, cavities, patchy consolidations, pulmonary infiltrates, or pleural abnormalities. Computed tomography (CT) provides greater diagnostic detail by identifying subpleural nodules, cystic or cavitary lesions, focal consolidations, linear migration tracts, pleural effusions, and pleural thickening [3,7,8]. Although none of these imaging findings are pathognomonic, a definitive diagnosis requires demonstrating characteristic *Paragonimus* eggs in sputum, stool, bronchoalveolar lavage fluid, pleural fluid, or lung tissue [2]. The sensitivity of microscopic examination is limited because egg excretion may be intermittent and is frequently absent during early infection or in patients with a low parasite burden.

Peripheral blood eosinophilia is a common laboratory finding, occurring in approximately 65–80% of patients with pulmonary paragonimiasis. Eosinophilic pleural effusions and elevated serum immunoglobulin E (IgE) concentrations are also common [2]. Serological assays detecting antibodies against *P. westermani* may support the diagnosis when parasitological examinations are negative; however, these tests cannot reliably distinguish active infection from previous exposure and may fail to detect antibodies against other *Paragonimus* species [2]. Furthermore, serological testing is not routinely available in many endemic settings, including Ecuador. Molecular methods are valuable for species identification but are currently used primarily for epidemiological and taxonomic investigations rather than routine clinical diagnosis [6].

Histopathological examination of chronic pulmonary lesions stained with hematoxylin and eosin (H&E) typically demonstrates eosinophil-rich chronic granulomatous inflammation accompanied by lymphocytes, histiocytes, multinucleated foreign body-type giant cells, variable epithelioid granuloma formation, hemorrhage, and fibrosis. *Paragonimus* eggs may appear intact, degenerated, fragmented, or calcified and are frequently surrounded by granulomatous inflammatory reactions. Within lung tissue, the eggs are characteristically round to ovoid, yellowish-brown, thick-shelled, and operculated at one pole, with reported dimensions ranging from approximately 80–120 μm in length and 45–70 μm in width. Under polarized light microscopy, the eggshell may exhibit birefringence [9,10,11].

Praziquantel is the treatment of choice for pulmonary paragonimiasis and is commonly administered orally at a dose of 25 mg/kg three times daily. Triclabendazole, administered as two oral doses of 10 mg/kg on the same day, represents an effective alternative therapy [2]. Triclabendazole is unavailable in Ecuador.

We report pulmonary paragonimiasis in an adolescent from the Ecuadorian Amazon presenting with recurrent hemoptysis and a solitary mixed solid-cystic lung lesion. Despite repeatedly negative parasitological examinations, the diagnosis was established by histopathological identification of *Paragonimus* eggs in the surgically resected lesion, highlighting the diagnostic value of histopathology when conventional investigations are inconclusive.

## 2. Case Presentation

A 17-year-old mestizo female, born and residing in the northern Ecuadorian Amazon (Lago Agrio, Sucumbíos Province), was admitted to the Department of Pulmonology of a tertiary referral hospital in Quito after transfer from her provincial hospital because of a 5-year history of cough with recurrent blood-tinged sputum, with increasingly frequent and severe episodes during the preceding months.

Approximately one year before referral (2024), she had received several courses of antibiotics at the referring hospital without clinical improvement. There, pulmonary tuberculosis was investigated using GeneXpert MTB/RIF assays software version 4.7b and mycobacterial cultures, all of which yielded negative results. Stool microscopy identified *Ascaris lumbricoides* and *Entamoeba coli*, for which albendazole was administered. Direct microscopic examination of fresh sputum was negative for *Paragonimus* eggs. A non-contrast chest computed tomography (CT) scan demonstrated a solitary cavitary lesion in the apical region of the left lung. Because of persistent hemoptysis, she was referred to Quito for further specialist evaluation.

On admission (April 2025), the patient was afebrile and denied chest pain or dyspnea. She was alert, oriented, and hemodynamically stable. Her blood pressure was 115/65 mmHg, heart rate 82 beats/min, respiratory rate 18 breaths/min, oxygen saturation 92%, and body weight 38 kg. Physical examination revealed a symmetric chest with preserved expansion and normal bilateral vesicular breath sounds without adventitious sounds. No lymphadenopathy, peripheral edema, or focal neurologic deficits were identified. The remainder of the physical examination was unremarkable.

During a detailed epidemiological interview, the patient reported frequent consumption of raw freshwater crabs and freshwater shrimp collected from a river near her house. Her medical history was significant for epilepsy diagnosed at 8 years of age, for which she was receiving carbamazepine and clonazepam. She denied tobacco, alcohol, or illicit drug use. Because of her history of epilepsy, the patient underwent neurologic evaluation. Electroencephalography showed age-appropriate activity, and brain magnetic resonance imaging revealed no structural abnormalities.

Flexible bronchoscopy with bronchoalveolar lavage revealed no endobronchial lesions, and microscopic examination did not identify *Paragonimus* eggs or other parasitic structures. CT pulmonary angiography did not demonstrate vascular malformations that could account for the hemoptysis. Non-contrast and contrast-enhanced chest CT demonstrated persistence of a solitary lesion in the apical region of the left lung (Figure 1).

Hematologic testing demonstrated peripheral eosinophilia, with an absolute eosinophil count of 3.68 × 10^3^/µL or 31.2% of the total leukocyte count (normal range: 0–0.4 × 10^3^/μL or 30–350 cells/μL). Serum immunoglobulin E was elevated at 1100 IU/mL (normal range for adults: 0–100 IU/mL). Her metabolic panel was normal. Cytologic examination of the bronchoalveolar lavage fluid was negative for malignant cells and showed chronic inflammation with a predominance of histiocytes; no parasitic structures were identified. Periodic acid–Schiff, Grocott methenamine silver, and Ziehl–Neelsen stains were negative, and mycobacterial cultures showed no growth. Autoimmune evaluation showed negative anti-cyclic citrullinated peptide antibodies, a positive immunoglobulin M rheumatoid factor, and negative anti-SS-B antibodies.

Because the pulmonary lesion persisted on two CT scans performed approximately 1 year apart (2024 and 2025), a CT-guided percutaneous lung biopsy was undertaken. Histopathologic examination was negative for malignancy and demonstrated fibroconnective tissue with chronic inflammation and liquefactive necrosis, but no specific etiologic diagnosis could be established. The patient subsequently underwent uniportal left video-assisted thoracoscopic surgery with resection of the left pulmonary lesion involving segments 1 and 2. Histopathologic examination by hematoxylin and eosin (H&E) staining—a standard laboratory method used in histology and pathology to color tissue sections so cellular structures are visible under a microscope—of the surgical specimen demonstrated chronic eosinophil-rich granulomatous inflammation, confluent epithelioid granulomas, and multinucleated foreign body-type giant cells. Several thick-shelled, yellow-brown, ovoid-to-elongated structures were identified, some with a visible operculum (Figure 2).

Following the histopathologic diagnosis of pulmonary paragonimiasis, praziquantel was administered orally at 25 mg/kg every 8 h for 3 consecutive days. At the 30-day post-treatment evaluation, the patient reported complete resolution of cough and hemoptysis. Follow-up chest radiography showed no residual pulmonary lesion, and the surgical wound healed without complications. The absolute eosinophil count decreased to 0.55 × 10^3^/µL (6.1% of the leukocyte count). During 1 year of follow-up (May 2026), she remained free of respiratory symptoms, and repeated microscopic examinations of sputum and stool specimens were negative for *Paragonimus* eggs.

## 3. Discussion

This case illustrates the diagnostic complexity of pulmonary paragonimiasis and highlights the value of histopathological examination of adequately sampled lung tissue when conventional parasitological investigations are repeatedly negative. The patient had several strongly suggestive features of pulmonary paragonimiasis, including years of recurrent hemoptysis, residence in an endemic region, consumption of raw freshwater crustaceans, marked peripheral eosinophilia, elevated serum IgE, and a persistent mixed solid-cystic pulmonary lesion. Nevertheless, the diagnosis was delayed for several years because the clinical and radiological findings were nonspecific and conventional examinations for *Paragonimus* eggs were negative. Failure to identify *Paragonimus* eggs in repeated sputum, stool, and bronchoalveolar lavage examinations did not exclude pulmonary paragonimiasis. Egg excretion may be intermittent and depends on parasite maturity, worm burden, communication of the parasitic cyst with the bronchial tree, and the timing and quality of specimen collection. In this patient, the small solitary subpleural lesion and probable low parasite burden may explain the repeatedly negative parasitological findings. Pulmonary paragonimiasis is frequently confused with tuberculosis and other chronic pulmonary diseases because clinical manifestations such as cough and hemoptysis, together with radiological findings including nodules and cavitary lesions, overlap with several infectious and tumoral conditions. This diagnostic challenge is particularly relevant in tropical endemic regions, where tuberculosis and paragonimiasis may coexist [3,4,5]. In the present case, repeated negative GeneXpert MTB/RIF assays, mycobacterial cultures, acid-fast staining, and fungal stains reduced the likelihood of tuberculosis and pulmonary mycosis. The persistence of a solitary necrotic pulmonary lesion also raised concern for malignancy, vascular malformation, or chronic localized infection. The investigations performed during hospitalization helped exclude these alternative diagnoses.

The epidemiological history was highly informative. The patient lived in the northern Amazon, an area where paragonimiasis is endemic [11,12] and reported consuming raw freshwater crabs and freshwater shrimp collected locally. This information underscores the importance of targeted dietary and environmental history-taking in patients from endemic regions who present with hemoptysis or unexplained pulmonary lesions. In Ecuador, *P. mexicanus* metacercariae have been documented in several freshwater crab species, particularly relevant to the present patient from the northern Ecuadorian Amazon; 37.1% (13/35) of *Trichodactylus faxoni* collected in 2013 were infected, while earlier Amazonian surveys reported infection in 96.1% of *Moreirocarcinus emarginatus* [3]. These findings demonstrate substantial enzootic transmission in freshwater crustaceans and support the epidemiological significance of consuming raw or insufficiently cooked crabs.

Marked peripheral eosinophilia was another important diagnostic clue. The absolute eosinophil count of 3.68 × 10^3^/µL together with a serum IgE concentration suggested a tissue-invasive helminth infection [2]. Although eosinophilia is not specific for paragonimiasis, its presence in a patient with hemoptysis, a pulmonary lesion, and relevant dietary exposure should prompt investigation for paragonimiasis. The marked decline in the eosinophil count after praziquantel and surgical removal of the lesion provided additional indirect support for pulmonary paragonimiasis. In this patient, the RF-IgM positivity may therefore have represented a nonspecific reactive phenomenon associated with the longstanding *Paragonimus*-induced pulmonary granulomatous inflammation, although an incidental finding cannot be excluded. In the absence of compatible clinical features, it was not considered evidence of clinically significant autoimmune disease.

The radiological findings were compatible with pulmonary paragonimiasis but were not specific for the disease. The lesion was solitary, subpleural, and mixed solid-cystic, with a necrotic center and peripheral contrast enhancement (Figure 1). Such findings may reflect chronic inflammation, parasite-induced tissue injury, fibrosis, or formation of a parasitic cyst. Pulmonary paragonimiasis may manifest as nodules, cavities, consolidations, pleural effusions, or linear migration tracts [3,7,8]. The persistence of the lesion supported a chronic localized process. Because these imaging findings overlap with those of tuberculosis, abscesses, granulomatous infections, and neoplasms, tissue diagnosis was ultimately required.

Importantly, the nondiagnostic result of the CT-guided percutaneous biopsy illustrates a limitation of small tissue samples: focal eggs or characteristic granulomatous areas may be missed, particularly when parasitic elements are sparsely distributed within a heterogeneous lesion. In contrast, surgical excision provided sufficient tissue to demonstrate multiple operculated eggs and the associated granulomatous inflammatory response [11]. Therefore, when a persistent pulmonary lesion remains unexplained, surgical biopsy may be considered. In this patient, histopathological examination of the resected lesion established the diagnosis by demonstrating characteristic *Paragonimus* eggs with a visible operculum.

A therapeutic consideration in this patient was the concomitant use of carbamazepine for epilepsy. Carbamazepine is a potent hepatic CYP3A4 inducer and can increase the first-pass metabolism and clearance of praziquantel, markedly reducing its systemic exposure and potentially compromising therapeutic efficacy. Therefore, concomitant administration was carefully evaluated. Awareness of this interaction is particularly important when treating paragonimiasis in patients receiving chronic enzyme-inducing antiepileptic drugs.

Following surgical resection and praziquantel, the patient experienced complete resolution of cough and hemoptysis and a substantial decrease in peripheral eosinophilia. She remained asymptomatic during a one-year follow-up. The combination of characteristic operculated eggs, compatible granulomatous histopathology, epidemiological exposure, eosinophilia, and a favorable clinical course strongly supported the diagnosis of pulmonary paragonimiasis.

In clinical practice, the diagnosis of pulmonary paragonimiasis should integrate epidemiological exposure, clinical manifestations, eosinophilia, imaging findings, and parasitological, serological, histopathological, and, when available, molecular evidence. CT scanning can characterize pulmonary lesions but does not confirm the diagnosis. Definitive diagnosis is supported by detection of *Paragonimus* eggs in sputum, pleural fluid, or swallowed eggs in stool, although intermittent egg excretion may reduce sensitivity; BAL may also detect eggs, but a negative result does not exclude infection. Serology is supportive but may not distinguish active from previous infection, while histopathology can confirm the diagnosis when characteristic eggs are identified in adequately sampled tissue. PCR may enable sensitive detection and species identification but remains limited in routine clinical practice.

## 4. Conclusions

In conclusion, pulmonary paragonimiasis should be considered in patients from endemic areas presenting with chronic hemoptysis, eosinophilia, and persistent pulmonary lesions, even when conventional parasitological examinations are negative. A limitation of this study was the inability to identify the *Paragonimus* eggs observed in the lung tissue to the species level. A detailed history of freshwater crustacean consumption and histopathological examination of adequately sampled lung tissue may be crucial for diagnosis. Antiparasitic therapy is generally sufficient, whereas surgical resection should be reserved for selected cases with complications, persistent lesions, or unresolved diagnostic uncertainty.

## Figures and Tables

**Figure 1:**
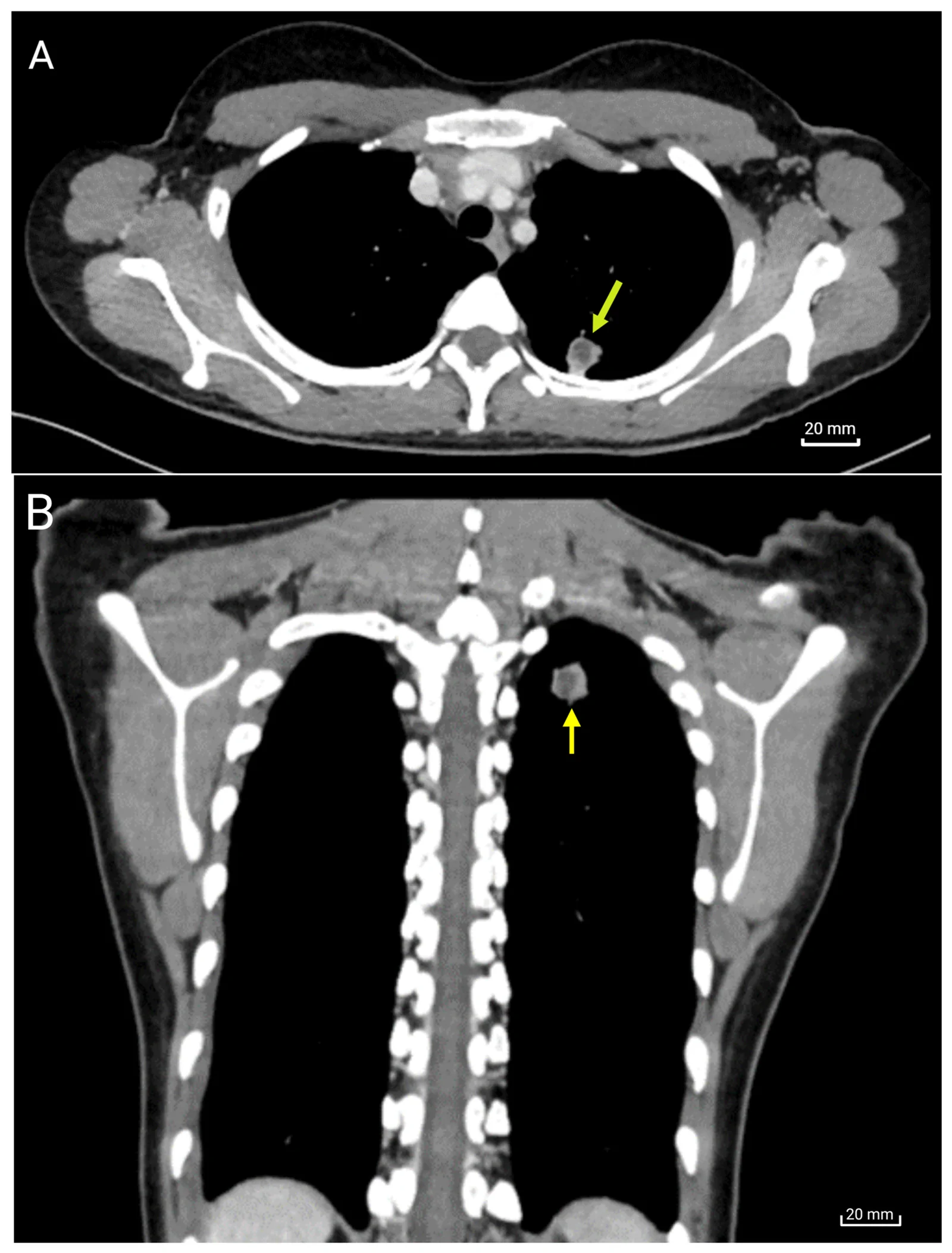
Chest CT scan findings. (**A**) Axial image showing a solitary subpleural mixed solid-cystic lesion in the apical region of the left upper lobe. (**B**) Coronal CT reconstruction demonstrates the same lesion as in (**A**). The lesion shows a central hypodense component and peripheral enhancement, measuring approximately 12 × 13 mm (yellow arrow). No pleural effusion or significant mediastinal lymphadenopathy was observed.

**Figure 2:**
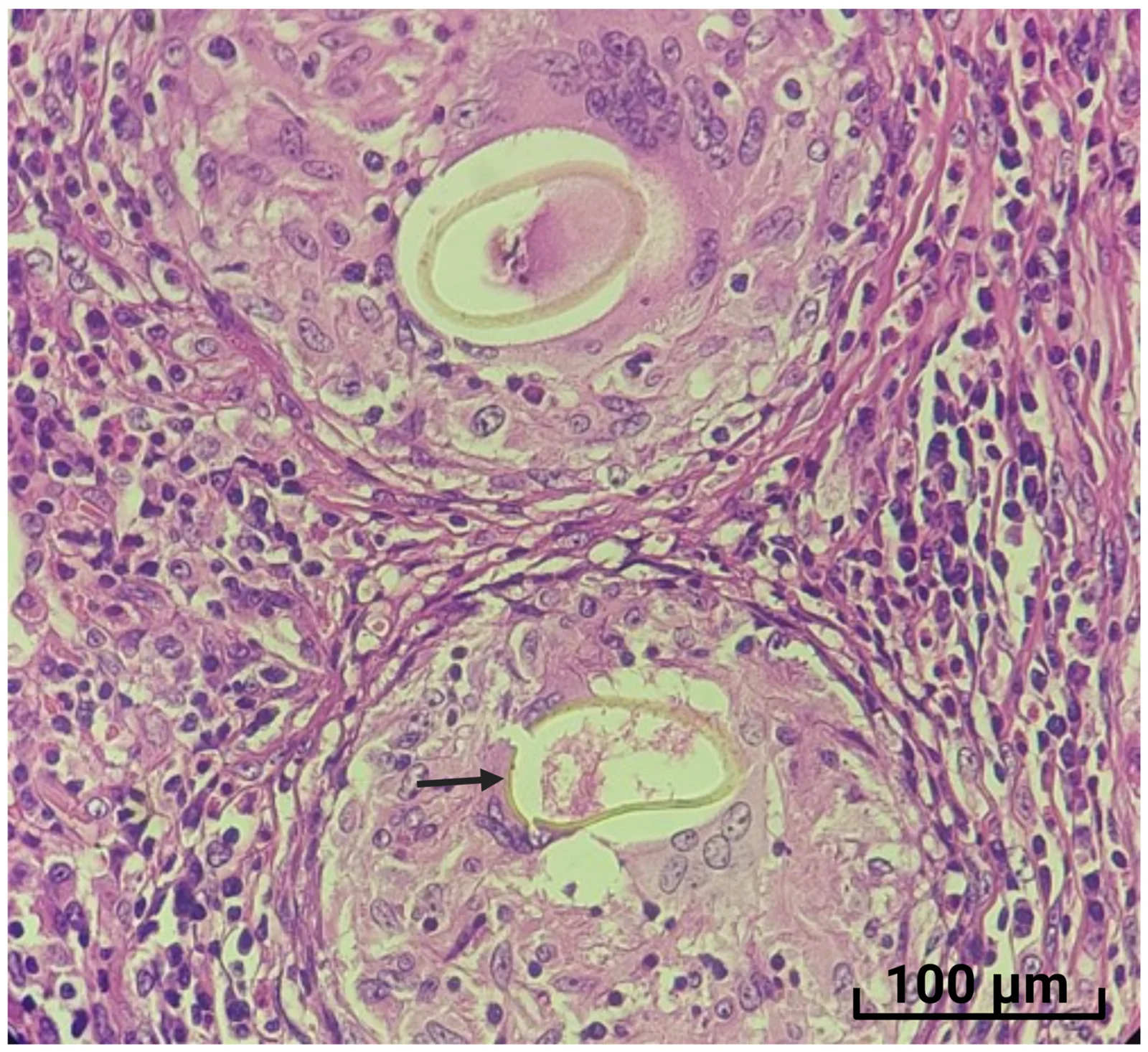
Histopathological findings in the resected pulmonary lesion. H&E staining demonstrates two ovoid-to-elongated, thick-shelled, yellow-brown structures measuring approximately 80–100 µm × 45–55 µm. An operculum is visible at one pole of the lower egg (arrow). Their size and morphology are consistent with *Paragonimus* spp. eggs. The eggs are surrounded by lymphohistiocytic granulomatous inflammation with abundant eosinophils. Confluent epithelioid granulomas and multinucleated foreign body-type giant cells are also present (×40). Species-level identification was not performed.

## Data Availability

Data are freely available.
